# Influence of Ethnolinguistic Diversity on the Sorghum Genetic Patterns in Subsistence Farming Systems in Eastern Kenya

**DOI:** 10.1371/journal.pone.0092178

**Published:** 2014-03-17

**Authors:** Vanesse Labeyrie, Monique Deu, Adeline Barnaud, Caroline Calatayud, Marylène Buiron, Peterson Wambugu, Stéphanie Manel, Jean-Christophe Glaszmann, Christian Leclerc

**Affiliations:** 1 UMR AGAP, CIRAD, Montpellier, France; 2 National Genebank of Kenya, KARI, Nairobi, Kenya; 3 UMR DIADE, IRD, Montpellier, France; 4 UMR LPED, Université Aix-Marseille/IRD, Marseille, France; 5 UMR AMAP, CIRAD, Montpellier, France; New York State Museum, United States of America

## Abstract

Understanding the effects of actions undertaken by human societies on crop evolution processes is a major challenge for the conservation of genetic resources. This study investigated the mechanisms whereby social boundaries associated with patterns of ethnolinguistic diversity have influenced the on-farm distribution of sorghum diversity. Social boundaries limit the diffusion of planting material, practices and knowledge, thus shaping crop diversity *in situ*.

To assess the effect of social boundaries, this study was conducted in the contact zone between the Chuka, Mbeere and Tharaka ethnolinguistic groups in eastern Kenya. Sorghum varieties were inventoried and samples collected in 130 households. In all, 297 individual plants derived from seeds collected under sixteen variety names were characterized using a set of 18 SSR molecular markers and 15 morphological descriptors. The genetic structure was investigated using both a Bayesian assignment method and distance-based clustering. Principal Coordinates Analysis was used to describe the structure of the morphological diversity of the panicles. The distribution of the varieties and the main genetic clusters across ethnolinguistic groups was described using a non-parametric MANOVA and pairwise Fisher tests.

The spatial distribution of landrace names and the overall genetic spatial patterns were significantly correlated with ethnolinguistic partition. However, the genetic structure inferred from molecular makers did not discriminate the short-cycle landraces despite their morphological distinctness. The cases of two improved varieties highlighted possible fates of improved materials. The most recent one was often given the name of local landraces. The second one, that was introduced a dozen years ago, displays traces of admixture with local landraces with differential intensity among ethnic groups. The patterns of congruence or discordance between the nomenclature of farmers’ varieties and the structure of both genetic and morphological diversity highlight the effects of the social organization of communities on the diffusion of seed, practices, and variety nomenclature.

## Introduction

Identifying factors involved in crop evolution is of great importance for genetic resource conservation and crop improvement. Crop genetic diversity patterns result from selection, migration and genetic drift processes which are strongly influenced by human action. Recent studies combining linguistic, archeological and genetic data have unraveled the past domestication and diversification processes of crops such as banana [1] and sweet-potatoes [2], on a large time-space scale, by linking global diversity patterns to human migrations. However, the evolution of crops is still ongoing in smallholder farming systems under the pressure of agro-ecological conditions and farmers’ management practices [3]. The study of these processes at the community scale is complementary to large time-space approaches and contributes to the general understanding of the *in situ* genesis of crop genetic patterns.

Social boundaries contribute to the evolution of crop populations both directly, by determining seed flows, and indirectly, by inducing the divergence of seed selection practices [4]. Previous studies notably showed that the ethnic organization of farming communities plays an important role in differentiating the domesticated populations of allogamous crops [5], vegetatively-propagated crops [6] and animals [7].

Sorghum (*Sorghum bicolor* L. Moench) is an annual cereal extensively cultivated in smallholder farming systems because of its ability to grow under harsh climatic conditions. De Wet and Huckabay [8] and Harlan et al. [9] suggested that the spatial distribution of sorghum botanical races in Africa was related to that of the ethnic groups, but this hypothesis was not further tested. In a study undertaken in Niger, Deu et al. [10] suggested that human ethnic diversity has probably a greater impact on sorghum diversity than recent environmental constraints. However, the authors were not able to assess this hypothesis as the spatial localization of the different ethnic groups in Niger corresponded to different agro-ecological regions. Thus, deciphering how the social organization of farmers affects the structure of sorghum diversity remains a challenge.

This article addresses the role of social boundaries in sorghum evolution and diversification processes. It set out to identify the mechanisms whereby social boundaries, associated with ethnolinguistic diversity patterns, shape sorghum genetic diversity on-farm. To study only the main effect of social boundaries, this study focused on an ethnolinguistic contact zone where both geographical distance between ethnic groups and agro-ecological variability were limited. If social boundaries do not limit seed-mediated gene flows and the diffusion of selection practices, then no relation should be observed between ethnic diversity patterns and both the genetic and morphological structure of sorghum diversity. Otherwise, it would reflect the impact of social boundaries on the evolutionary mechanisms that shape sorghum diversity *in situ*.

Farmers’ varieties are relevant units for studying on-farm crop diversity as they are consciously defined and named by farmers for management, selection, seed exchanges and knowledge transmission purposes [11]. Farmer’s nomenclature and taxonomy of crop varieties is a marker of knowledge diffusion and exchanges across communities [12], while the distribution of the genetic and morphological diversity of crop populations reflects gene flows and selection forces [5]. This study thus used molecular markers to estimate genetic diversity and compared the spatial distribution of varieties with genetic spatial patterns according to ethnic groups. These patterns were then discussed regarding the congruence between farmer’s varieties and the structure of their genetic and morphological diversity. Combining these three approaches enabled us to investigate the influence of social boundaries on the evolutionary mechanisms that shape sorghum diversity *in situ*.

Clarifying the effect of social boundaries on crop evolutionary mechanisms has important applications for crop genetic resource collection, characterization and conservation. This study hence contributes to increasing the overall understanding of on-farm crop diversification processes. By highlighting the overall role of societies in shaping crop diversity, it stresses the relevance of multidisciplinary approaches for crop genetic diversity studies.

## Materials and Methods

### Ethics statement

This was a collaborative study between CIRAD and KARI-National Genebank of Kenya. KARI has the national mandate for the collection and conservation of all plant genetic resources and documentation of all accompanying information. Under this framework and mandate, the study was mounted and all laid down institutional and administrative procedures were carefully followed prior to undertaking the study. Based on the aforementioned mandate given to KARI, no specific permission was required to undertake the study. Though KARI does not have a body designated as ethical review board, it has equivalent committees and administrative organs which review proposed research activities before granting approval. Research clearance was therefore sought from these organs at all levels including the institutional legal office. Local government administrative as well as agricultural extension officers were informed of the study and kept updated of the activities.

During the survey, the mandate given to KARI as well as the importance of the study, both nationally and globally, was explained to the farmers and concurrence was sought before undertaking the study activities. According to KARI’s procedures governing genetic resources collection and documentation, prior informed consent was obtained verbally and not recorded, all with the understanding that the process would only involve collection of genetic resources and no sensitive traditional knowledge. Where such consent was not granted, the germplasm collectors stopped any more activities in that particular household. In each household, we interacted with the female household head. Upon granting consent, they were interviewed mainly on their ethnicity and the sorghum varieties they grew. The survey was conducted by the authors among them V.L, A.B, P.W, and C.L with questions being translated by a local field assistant. We confirm that sorghum, the studied crop, is neither endangered nor protected.

### Study site: Agro-ecological conditions and ethnic organization

**Figure 1:**
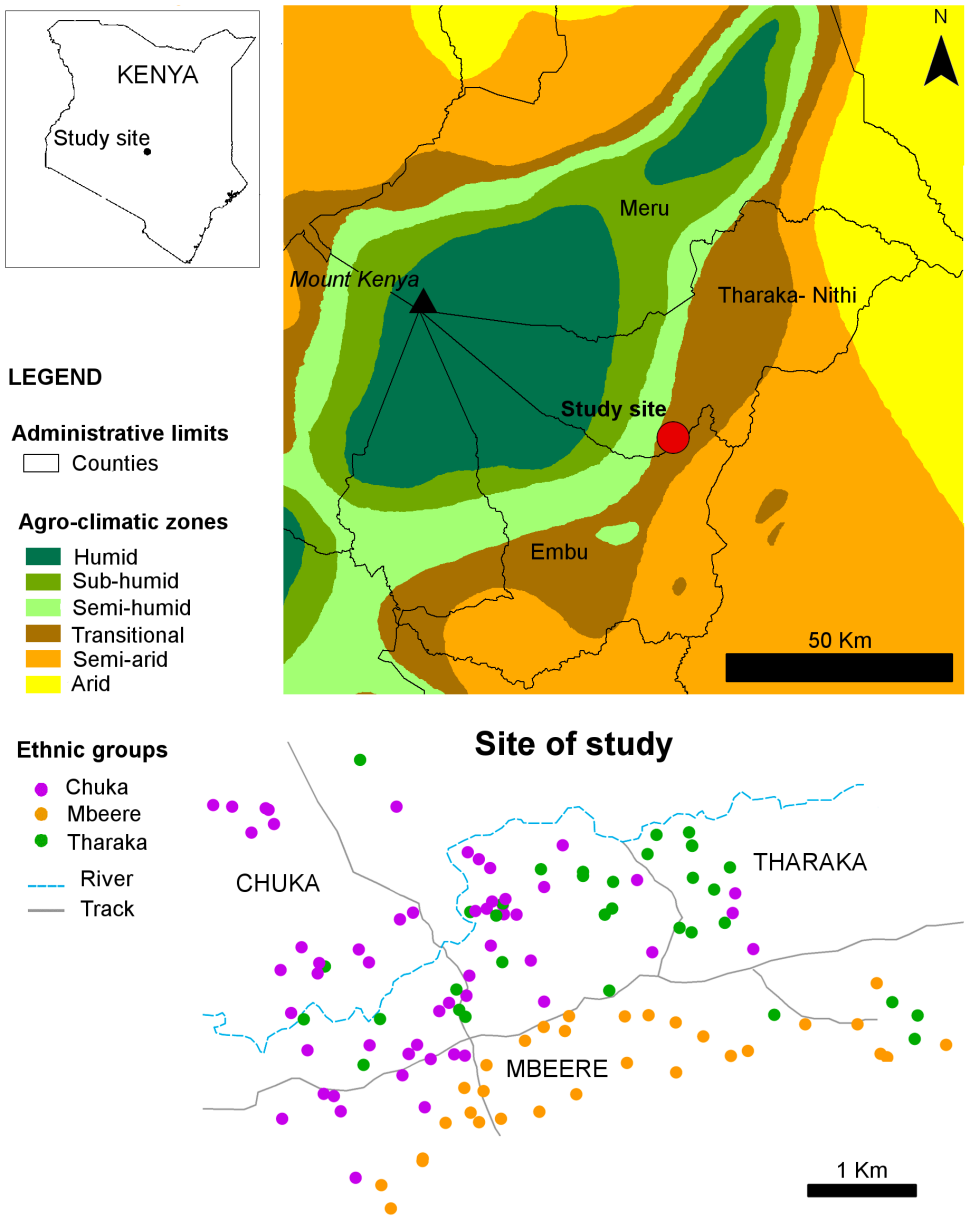
Study site location. Map of the eastern side of Mount Kenya and location of the farms where sorghum samples were collected (colors correspond to the ethnic identity of the male house-head).

This study was conducted on the eastern slope of Mount Kenya (0°24'27.88"S, 37°46'35.59"E), in an ethnolinguistic contact zone between Chuka, Tharaka and Mbeere groups (Figure 1). The three ethnolinguistic groups (hereafter ethnic) live within the same agro-ecological zone, as defined by Jaetzold et al. [13]. The study site was 15 km-square, and the elevation ranged from 810 to 946 m above sea level, so rainfall and temperature variability was limited. The mean temperature on the area ranges between 21.7°C and 23.9°C. The mean rainfall is about 700–800 mm per year, distributed across two rainy seasons with the Long Rains occurring from March to May and the Short Rains from October to December [14]. Soil characteristics are homogeneous in the area occupied by the three ethnic groups, corresponding to well drained Ferralsols, with a loamy-sand texture and moderate fertility [13].

The three ethnic groups, Chuka, Tharaka and Mbeere, migrated to the study area by the end of the 19^th^ century, either because of a population increase or because of recurrent drought [15]. Social boundaries exist between Chuka, Tharaka and Mbeere groups as revealed by their distinct ethnic identity, and their current cultural and linguistic differences [16], [17]. The Mbeere are closely related to the Embu group [18], while the Chuka and Tharaka are related to the Meru group. The Mbeere and Chuka had conflictual mutual relationships in the past [19], while the Chuka and Tharaka maintain strong social ties and consider they are kin [20]. Intermarriage is usual between the Chuka and Tharaka, while it is very uncommon between the Mbeere and Chuka or Tharaka (unpublished data). Men usually settle near their father’s compound once they get married. The residence is thus patrilocal [16]. The three ethnic groups present a non-random spatial distribution. The Mbeere households are located in the southern part of the study area, the Tharaka mostly on the north-eastern side, and the Chuka on the north-western side (Figure 1). Consistently with the social relationships between groups depicted above, a clear spatial boundary was found between the Mbeere and both the Chuka and Tharaka, while the Chuka and Tharaka appeared to be spatially more mixed.

The three ethnic groups manage low-input cropping systems that harbor high specific and infra-specific crop diversity. Cropping systems are based on cereals and legumes that are usually intercropped. Sorghum (*Sorghum bicolor*), cowpeas (*Vigna unguiculata*), maize (*Zea mays*), mungo bean (*Vigna radiata*) and pearl millet (*Pennisetum glaucum)* are the main crop species grown in the area. Sowing is done either by hand-dibbling or by drilling, while plowing is done with animals. The different sorghum varieties are either grown in separate plots or mixed together within farmers’ fields. Improved varieties, mainly disseminated by the extension services of the Kenyan Ministry of Agriculture, have also been adopted by the farmers. They are cultivated together in the same field with the local varieties (or landraces). Farmers distinguish between short-cycle varieties that can be grown either from October to January or from March to June, and long-cycle varieties that are subjected to the ratooning practice [21] (Figure S1). These long-cycle varieties are sown in October, the vegetative part being cut before the grains are mature to stimulate regrowth from basal buds, and panicles are finally harvested in July.

### Data collection

#### Sorghum inventory and germplasm collection

The field work consisted of two stages. A preliminary survey was carried out to estimate the frequency of varieties in the three ethnic groups. The strategy for on-farm germplasm collection was then based on that estimation of diversity, as it aimed at representing the diversity and frequencies of each variety in each ethnic group.

The preliminary inventory survey was conducted in both January (Short Rains cropping season) and June 2011 (Long Rains cropping season), just before harvesting and prior to germplasm collection. The inventory of sorghum varieties was based on the local names as reported by women farmers who were in charge of sorghum selection in each of the 124 households surveyed. Indeed, grain crop farming comprising seed sowing, harvesting, selection and trading is ensured by women ([16], personal observation). The ethnolinguistic identity of male house-heads or single women was also recorded, women becoming members of the family of their husband when they get married in this patrilinear society.

Sorghum panicles were then collected from a total of 130 households selected randomly. In 22 of these households, panicles were collected in both January and July, while the rest of the households were visited only in January (34 households) or in July (74 households). Half of these 130 households were visited during the preliminary survey described above. They represented about half of the total number of households in the area, hence insuring a good representativeness. 60 households belonged to the Chuka ethnic group, 35 to the Mbeere and 35 to the Tharaka. In order to be representative of the sorghum population of each ethnic group, all the varieties grown in each household were collected, except a highly dominant variety of improved origin (*Kaguru*). As this variety was much more abundant than the others, we limited the number of samples collected. We thus sampled *Kaguru* variety randomly in a maximum of 19 households per ethnic group. One or two individual panicles of each variety were collected in each household cultivating it. The mean number of varieties collected per household was 1.5 (min: 1, max: 6). It was similar across ethnic groups, as well as the mean number of panicles of each variety sampled per household (Table S1). The fraction of households where each variety was collected for the study of genetic diversity was correlated to the fraction of households where each variety was previously inventoried (Linear regression R^2^: 0.77, Figure S2). In all, 290 samples were collected on-farm after harvest, each consisting of a single panicle. About 47% of the individual plants were sampled from the Chuka ethnic group, 30% from the Tharaka and 23% from the Mbeere. Information concerning the names, the origin (local or improved) and the cycle length of each sampled panicle was recorded from women house-heads, and we recorded the geographic coordinates of each household using a global positioning system (GPS).

#### DNA extraction and SSR genotyping

Seeds from the 290 panicles collected on-farm were sown in an experimental field *in-situ*, and the leaves of one sibling randomly chosen for each mother plant were collected and stored on silicagel. Leaves from seven individuals grown from certified seeds of the improved varieties *Serendo* and *Gadam* were also collected as controls. In total 297 individual plants were thus used for the genetic diversity study.

Twenty-two pairs of primers were selected for their high polymorphism in central Kenya (unpublished data) and West Africa [10]; twenty of them were part of a set of reference microsatellite markers proposed by Billot and colleagues ([22], http://sat.cirad.fr/sat/sorghum_SSR_kit/). Loci were distributed over the 10 chromosomes. DNA was extracted from dried leaves and the polymerase chain reaction amplifications were done following the procedure described previously [10], [22]. The fluorescent dye–labeled PCR products from differentially labeled primers and with non-overlapping size were pooled and subjected together to capillary electrophoresis using a 24-capillary 3500xL System (Applied Biosystems). GeneMapper v 4.1 (Applied Biosystems) was used for genotype scoring. GeneScan 600 LIZ Size Standard v2.0 was added to each well, and three control samples were used to facilitate allele scoring [22]. Genotyping was done at the Montpellier Languedoc-Roussillon Genopole platform located on the CIRAD campus in Montpellier (France).

Four markers presenting either a high number of missing data, or low polymorphism (at a 99% threshold) were discarded from the analysis, so eighteen markers were kept, covering 9 chromosomes out of 10. The percentage of missing data for the 18 markers kept was 1%.Table S2 provides a list of these 18 markers and their description.

#### Panicle morphological characterization

Fifteen qualitative morphological traits were measured on the panicles of the 297 individuals that were genotyped (Table S3). Eight morphological descriptors were selected from the IPGRI descriptors [23] and were completed by seven additional descriptors for seeds and glumes characteristics that showed variability on the sorghum collected in our study area. Descriptors covered the characteristics of the whole panicle (panicle shape), seeds (color, presence of sub-coat, pericarp thickness, shape, endosperm texture and shattering) and glumes (color, adherence, covering, opening, texture, hairiness, awning and transversal wrinkle). Only qualitative traits were kept for these analyses because they are stable characteristics on which farmers base their nomenclature and classification [24]. Multiple characterizations of randomly sampled individuals enabled to check for morphological trait scoring consistency.

### Data analysis

#### Comparing sorghum assemblages between ethnic groups

We characterized each household by its sorghum assemblage, which is the panel of co-occurring sorghum varieties that are cultivated by the household. The differentiation of sorghum assemblages across ethnic groups was tested using a non-parametric Multivariate Analysis of Variance (perMANOVA, [25]). The PerMANOVA was implemented under the *adonis* function in the R package *vegan* [26]. The presence/absence matrix for sorghum varieties in each household was transformed into a distance matrix using the Bray-Curtis index [27]. The *adonis* function partitions the distance matrix according to grouping factors (ethnic groups) and compares the sum of squared distances within groups (which is the sum of squared distances from individual replicates to their group centroid) and between groups (which is the sum of squared distances from group centroids to the overall centroid). A pseudo F-ratio is then computed and compared to its distribution under the null hypothesis simulated using 4000 random permutations of the raw data. Pairwise Fisher exact tests implemented in the R package *fmsb* [28] were then used to compare the occurrence frequencies of the most frequent varieties across the Chuka, Mbeere and Tharaka ethnic groups. The calculation of p-values was corrected for multiple comparisons using the False Discovery Rate (FDR) procedure [29] implemented in the *p.adjust* function.

**Genetic diversity and genetic structure of sorghum populations. Genetic diversity within sampling populations.** The genetic diversity of sorghum populations sampled in each ethnic group was assessed using several indexes. The observed number of alleles and the observed heterozygosity were calculated using GENETIX 4.05.2 software [30]. The allelic richness corrected for sample size [31], the unbiased gene diversity (expected heterozygosity) corrected for small sample size [32], and the *F*_IS_ [33] of multi-locus genotypes were estimated using the procedures implemented in FSTAT 2.9.3.2 software [34]. These indexes were compared among ethnic groups using paired Pairwise Wilcoxon tests with False Discovery Rate (FDR) correction implemented in R (package *stats, pairwise.wilcox.test* function).

**Genetic structure assuming sampling populations.** Pairwise *F*_ST_ [33] were computed among the sorghum populations collected in the three ethnic groups. The significance of the differences was assessed using a permutation test (3000 permutations) and corrected using a Bonferroni procedure [35]. A multilocus G-test of differentiation, known to be accurate for measuring the genetic differentiation between populations with unbalanced sizes [36], was used to test the genetic differentiation between the populations sampled in each ethnic group (10000 permutations). Calculations were carried out using FSTAT 2.9.3.2. Pairwise G-tests implemented in GENEPOP 4.2 [37] were used to estimate the genotypic differentiation among pairs of populations and p-values were corrected for multiple tests using FDR correction (*p.adjust* function in the R package *stats*).

**Analysis at individual level.** Two complementary approaches, Bayesian clustering and Neighbor-Joining tree, were used to assess the genetic structure without defining a-priori populations. First, the genetic structure of sorghum populations was characterized using the Bayesian clustering algorithm implemented in STRUCTURE 2.3.3 software [38] and run on the Bioportal server (http://www.bioportal.uio.no). The admixture model with correlated allele frequencies was used, assuming that the genome of each individual resulted from the mixture of *K* ancestral populations. The estimated proportions of each individual’s genotype originating from each of the *K* ancestral populations (*q*) was calculated for *K* ranging from 2 to 10 ancestral populations (or clusters), with twenty runs for each *K* value. The burn-in period was set at 500 000 and 1 000 000 iterations were performed. The criterion suggested by Evanno et al. [39], based on the rate of change in the log probability of data between successive *K* values, was used to determine the most likely number of clusters (*K*). Second, a Neighbor-Joining tree [40] was built from a simple matching genetic dissimilarity index [41] using Darwin V5 software [42]. The results of both the Bayesian clustering and Neighbor-Joining methods were then compared to check for the consistency of the clusters. This led to what we refer to as an MMb (molecular-marker-based) classification scheme.

For further analysis, individuals whose estimated proportion of genome originating from one population (*q,* hereafter admixture coefficient) was below a 0.8 threshold were considered as resulting from admixture between the populations. Individuals whose *q* value was equal to or above 0.8 for a population were assigned to that population (hereafter cluster). To explain the MMb genetic structure, the assignment of individuals to clusters thus defined was crossed with information concerning their origin and cycle length as reported by farmers during the collection of samples *in situ*. The occurrence frequencies of each MMb genetic cluster were then compared across ethnic groups using Pearson's Chi-squared test, and pairwise Fisher exact tests with False Discovery Rate (FDR) correction for multiple comparisons.

To test a potential isolation-by-distance effect in cultivated sorghum, we applied Mantel test between pairwise genetic distances and geographical distances. The matrix of geographical distances among individuals was computed. The kinship coefficient of Loiselle et al. [43] was computed using SPAGeDI software [44] for each pair of cultivated sorghum individuals, producing a matrix of individual pairwise genetic distance. A stratified Mantel test implemented in the R package Vegan was used to test the significance of the correlation between the logarithm of the pairwise geographical distances [45] and sorghum individuals’ pairwise genetic relatedness. 4000 permutations of the locations of samples were done within the genetic clusters previously identified using STRUCTURE software (stratified test), as recommended by Meirmans [46] for populations presenting a strong genetic structure.

### Morphological structure of sorghum populations

To describe the structure of individual panicle morphological diversity, a dissimilarity matrix was computed on the basis of the 15 morphological traits coded through a total of 43 modalities using the simple matching index. The morphological similarity between individuals was then assessed using a Principal Coordinates Analysis (PCoA) using the R package *ade4*.

## Results

### Differences in variety assemblages across ethnic groups

**Figure 2:**
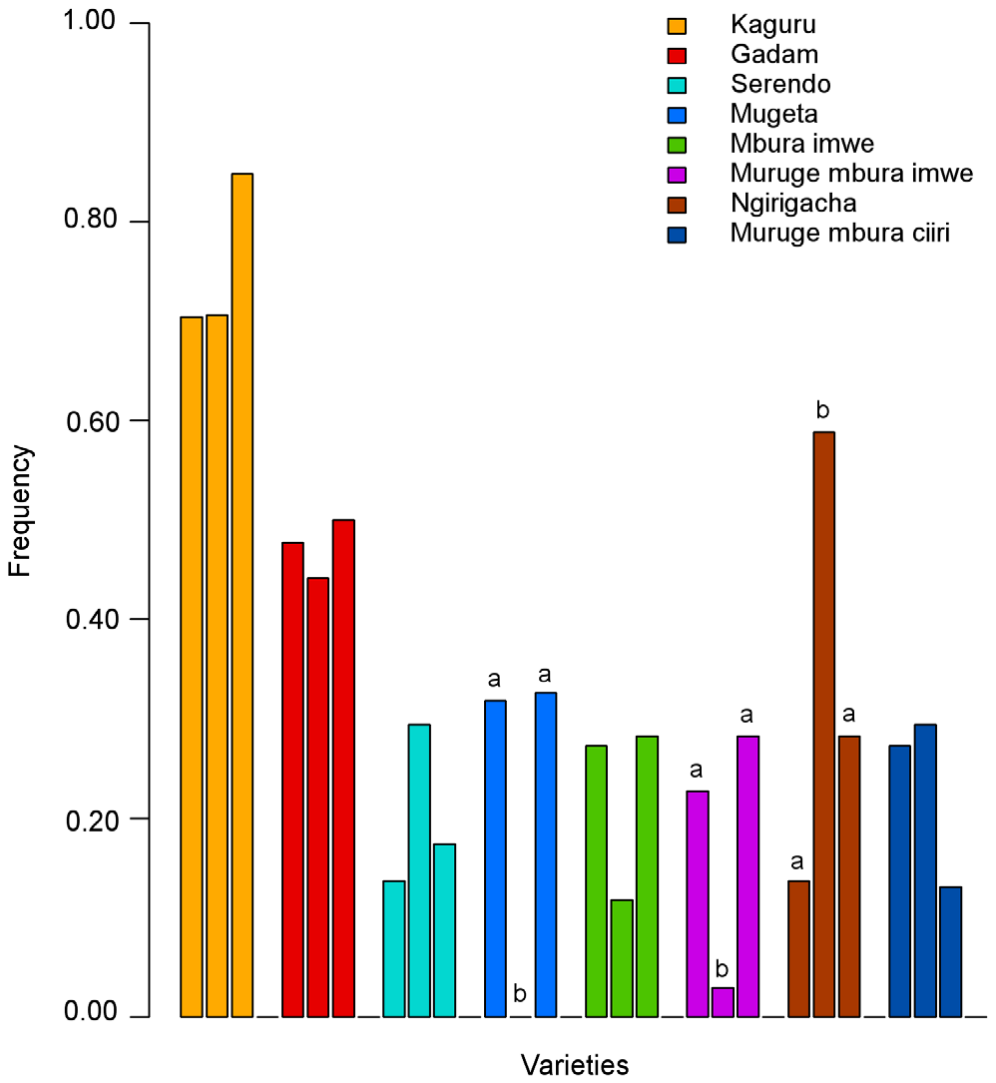
Frequency of the eight major varieties in each ethnic group. The vertical axis displays the percentage of farms where each variety was cultivated. Ethnic groups are present in the following order for each variety: Chuka, Mbeere, Tharaka. The letters (a, b) on top of the bars indicate the statistical significance of differences (Fisher test) at a 5% level after correction for multiple testing (FDR). For a given variety, ethnic groups with the same letter did not present significantly different frequencies.

On the basis of their local names, seventeen different varieties were inventoried among the 124 households visited during both the January and June surveys. 14 different varieties were respectively inventoried in the Chuka and Tharaka groups, and 10 in the Mbeere group, out of which 9 were shared by the three ethnic groups. The mean number of varieties inventoried in both cropping seasons per household was similar across ethnic groups (2.77, SE: 0.17 for the Chuka, 2.65, SE: 0.17 for the Mbeere, 3.02, SE: 0.21 for the Tharaka). The most frequent variety was *Kaguru,* (76% of the households), followed by *Gadam* (48% of the households), both of which are improved varieties. *Ngirigacha, Mugeta*, *Mbura imwe, Muruge mbura ciiri*, and *Muruge mbura imwe* were the most frequent local varieties (landraces) (Figure 2).

The non-parametric perMANOVA showed that sorghum variety assemblages differed significantly between ethnic groups (Table S4), even though the ethnic partition explained a limited part of variability (pseudo-F_2,121_ = 4.971, p-value  = 0.0002, R^2^ = 0.076). Pairwise Fisher exact tests confirmed that the frequency of three out of the five most frequent landraces differed significantly between ethnic groups, while the frequency of improved varieties (*Gadam*, *Kaguru* and *Serendo*) did not differ significantly between ethnic groups. *Muruge mbura imwe* and *Mugeta* were significantly less frequent in the Mbeere group than in the Chuka and Tharaka groups while *Ngirigacha* was significantly more frequent in the Mbeere group.

### Genetic and morphological structure of cultivated sorghum

**Figure 3:**
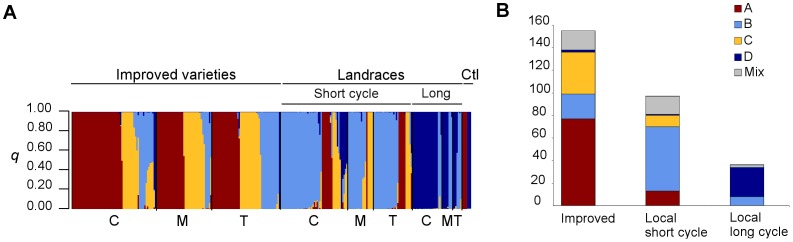
Genetic structure of the sorghum cultivated on the area of study. (A) Cluster assignment of 297 sorghum individuals estimated using STRUCTURE for *K* = 4. The genome of each individual is represented by a vertical line, which is partitioned into *K* colored segments that represent the admixture coefficient (*q*), i.e the estimated proportion of membership of its genome in each of the *K* clusters (Red: cluster A, light blue: cluster B, yellow: cluster C, dark blue: cluster D). Thick black lines separate the individuals identified by farmers as improved varieties, short-cycle landraces or long-cycle landraces, and control individuals (Ctrl), as labeled above the figure. Thin black lines separate individuals sampled in the different ethnic groups (Chuka: C, Mbeere: M, Tharaka: T, as labeled below the figure. The figure shown is based on the highest probability run at *K* = 4. (B) Number of individuals classified according to their origin and cycle length (farmers’ information) assigned to each MMb genetic cluster. The vertical axis indicates the number of individuals assigned to each cluster. Individuals were assigned to a cluster when their estimated admixture coefficient (*q*) for this cluster was equal to or over 0.8. Admixed individuals are represented in gray.

**Figure 4:**
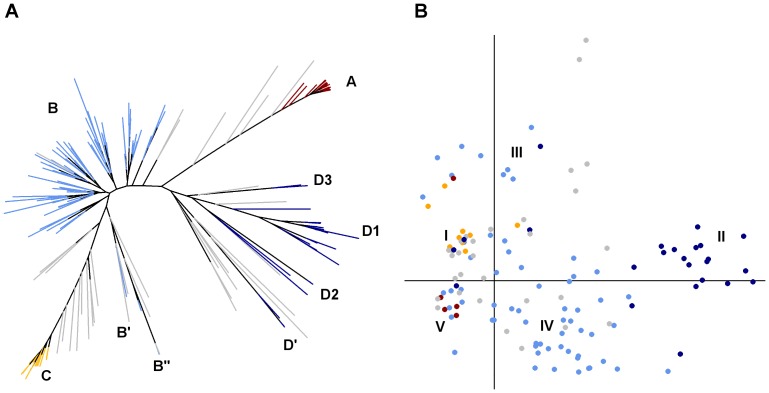
Genetic and morphological structure of the sorghum cultivated on the study area. (A) Neighbor-Joining tree based on 18 SSRs among sorghum plants using the simple matching index. Genetic clusters inferred by STRUCTURE are displayed using different colors (Cluster A: red, B: light blue; C: yellow, D: dark blue). Sub-clusters are identified by letters followed by a number. (B) Plot of the two first axes of the Principal Coordinates Analysis (PCoA) based on 15 panicle morphological traits using the simple matching index. The first axis (x) expresses 29.3*%* of the total variation and the second axis 13.2*%*. The main morphological groups are indicated by roman numerals and the MMb genetic assignment of individuals for *K* = 4 is displayed with the same colors as in figure 4.A.

The most likely number of populations (*K*) identified by STRUCTURE was *K* = 4. Indeed, the log-probability of data increased up to *K* = 4, where it reached a plateau. This was congruent with Evanno’s ΔK curve which presented a clear peak for *K* = 4. The populations (clusters) inferred by STRUCTURE for *K* = 4 (Figure 3.A) corresponded to distinct groups on the Neighbor-Joining tree (Figure 4.A). Cluster A and C were distinct and showed higher genetic uniformity than cluster B and D. Most of the individuals sampled (88%) showed an admixture coefficient (*q*) above or equal to *q* = 0.8, and they were thus assigned to the corresponding cluster. The remaining 12% of the individuals were considered to result from admixture between clusters.

The MMb genetic structure was found to be strongly related to the improvement status of the germplasm – improved varieties or local landraces, and by differences in growth-cycle length (Figure 3.B). Individuals assigned to the uniform clusters A and C were mostly improved varieties introduced by the extension services, while individuals assigned to the broader clusters B and D were mainly classified by farmers as local landraces. Moreover, almost all individuals assigned to cluster D were identified by farmers as long-cycle varieties (ratoon) while those individuals assigned to clusters A, B and C were mainly identified as short-cycle varieties.

Despite this global coherence, the characteristics of varieties reported by farmers showed some divergence from the MMb genetic classification. Twenty-two percent (22%) of the individual plants that were identified as long-cycle landraces by farmers during the collection were assigned to cluster B by STRUCTURE (Figure 3.B). A substantial proportion of individuals identified by farmers as short-cycle landraces were assigned to clusters A (13%) or C (10%). Indeed, young farmers may consider as local the varieties that were introduced a long time ago, perhaps before they began farming. Conversely, 14% of the individuals identified by farmers as improved varieties were assigned to cluster B.

The morphological diversity was summarized by the PCoA (Figure 4.B). The two first axes accounted for 29 and 13% of the variation, respectively. Axis 1 isolated a clear group on its positive side (II), corresponding to the major share of individuals assigned to MMb cluster D, while the rest of the individuals were broadly distributed along axes 1 and 2. Individuals assigned to MMb clusters A and C displayed narrow distributions indicating uniform morphological types, which is consistent with their improved origin and recent introduction. Individuals assigned to cluster C formed a distinct morphological group (I), discriminated on the third axis of the PCoA (expressing 10.7% of the total variation, data not shown). Individuals assigned to MMb cluster B displayed a broad distribution, reflecting high variability and continuous distribution across diverse morphotypes. It is noteworthy that some of the individuals assigned to MMb genetic clusters A and B displayed morphological similarity (Figure 4.B.), which may induce possible confusion in naming the recent improved variety and the local landraces (homonymy). Nevertheless, part of the individuals assigned to the MMb cluster B clustered in a separate morphological groups (III).

### Genetic differentiation of sorghum populations across ethnic groups

Various indexes were used to characterize the diversity displayed within each ethnic group (Table 1). The unbiased gene diversity estimates (*H_e_*) of Chuka and Tharaka sorghum populations were significantly higher than that of the Mbeere (Wilcoxon test: p-value < 0.01). Similar results were found for the unbiased allelic richness. *F_IS_* was very high in the three groups, yet it was significantly lower in the Chuka population as compared to those of both the Tharaka and the Mbeere (Wilcoxon test: p-value < 0.05 for both pairwise comparisons), in relation with the higher heterozygosity found within the Chuka sorghum population (0.033) compared to the other two populations (0.022 for the Mbeere and 0.023 for the Tharaka).

**Table 1:** Summary of the genetic polymorphism indexes of sorghum individuals sampled in each ethnic group.

Ethnic group	N_i_	N_hh_	*N_Al._*	*R_Al._*	*H_e_*	*H_o_*	*F_IS_*
**Chuka**	135	60	6.8	6.1^a^	0.590^a^	0.033	0.943^a^
**Mbeere**	68	35	4.7	4.6^b^	0.544^b^	0.022	0.959^b^
**Tharaka**	87	35	6.1	5.9^a^	0.569^a^	0.023	0.961^b^
**Total**	290	130	7.7	7.7	0.574	0.028	0.952

N_i_: number of samples, N_hh_: number of households, *N_Al_*_._: Mean number of observed alleles over the 18 loci, *R_Al_*_._: unbiased allelic richness corrected for sample size, *H_e_*: unbiased gene diversity, *H_o_*: observed heterozygosity, *F_IS_*: fixation index. The letters (a, b) next to the *R_Al_*, *H_e_* and *F_IS_* values indicate the statistical significance of their differences between ethnic groups (Wilcoxon test) at a 5% level after correction for multiple testing (FDR). For a given index, ethnic groups with the same letter did not present significant differences.

An exact G-test of genetic differentiation of sorghum across ethnic groups was significant (p-value  = 0.0205). The differentiation was clearer (G-test p-value  = 0.0026) when removing from the analysis the individuals assigned to cluster A, derived from the recent introduction of the *Gadam* improved variety. The Pairwise G-tests showed that genetic differentiation was highly significant between the sorghum populations of the three groups, being highest between the Chuka and both the Tharaka (p-value < 0.0001) and Mbeere (p-value  =  0.0002) populations and lowest between the Tharaka and Mbeere populations (p-value  =  0.0083). The *F_ST_* values between the sorghum populations of the three ethnic groups were low: 0.027 between the Chuka and Mbeere sorghum populations and 0.019 between the Chuka and Tharaka populations, both significant; and non significant between the Mbeere and Tharaka populations (*F_ST_* = 0.010).

**Figure 5:**
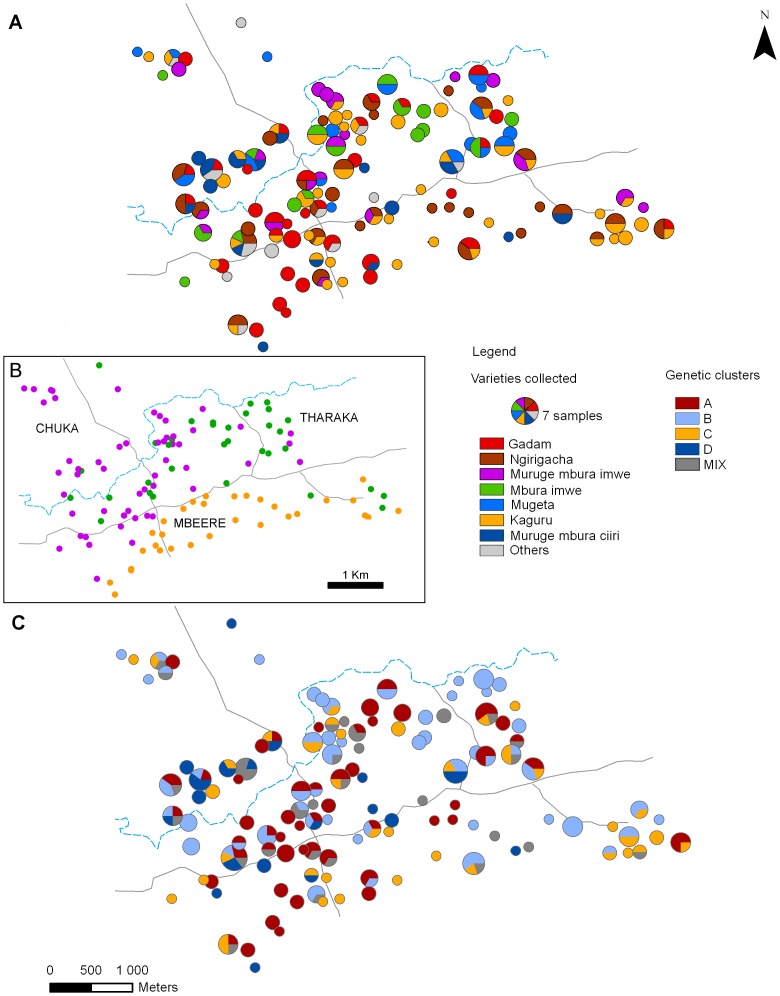
Spatial distribution of the sorghum varieties, ethnic groups, and sorghum genetic clusters. (A) Map of the named varieties collected in each ethnic group. Pie charts represent the number of samples of each variety collected in each household. The size of each circle is proportional to the number of individuals sampled. (B) Location of the ethnic groups (Purple: Chuka, Green: Tharaka, Orange: Mbeere). (C) Map of the number of sorghum individuals in each household assigned to each of the four MMb genetic clusters. Individuals were assigned to a cluster if their estimated genome fraction to that cluster, i.e. admixture coefficient (*q*), was higher than 0.8.

No significant relationship was found between the genetic relatedness of individuals and their geographical distance. The partial Mantel test was not significant (r = –0.38, p-value  = 0.130), and the correlogram did not display any significant spatial structure. Nevertheless, the spatial distribution of the four MMb genetic clusters was not uniform (Figure 5.C) and they were not evenly distributed across the three ethnic groups (Table 2). Pearson's Chi-squared test led to rejecting independence between the genetic clusters and the ethnic groups (p-value  = 0.003).

**Table 2:** Number of individuals sampled in each ethnic group and assigned to each MMb genetic cluster.

MMb cluster	Chuka	Mbeere	Tharaka	Total	Chi^2^	P-value
**A**	44 (33%) ^a^	21 (31%) ^a^	25 (29*%*) ^a^	90 (31%)	0.37	0.832
**B**	36 (27%) ^a^	18 (27%) ^a^	34 (39*%*) ^a^	88 (30%)	4.49	0.106
**C**	12 (9%) ^a^	17 (25%)^ b^	18 (21*%*) ^b^	47 (16%)	10.5	0.005
**D**	21 (15%)^ a^	5 (7*%*) ^ab^	4 (4*%*)^ b^	30 (11%)	7.7	0.021
**Mix**	22 (16%) ^a^	7 (10*%*) ^a^	6 (7*%*) ^a^	35 (12%)	4.7	0.097
**Total**	135 (100%)	68 (100%)	87 (100%)	290 (100%)		

Individuals with a *q* value equal to or above the threshold of 0.8 for a cluster were assigned to that cluster. The Chi-Square statistics and p-value compare, for each MMb cluster, the observed and the expected frequencies under the null hypothesis of independence. For each cluster, the letters indicate the statistical significance of the differences in its frequency between ethnic groups (Fisher test) at a 5% level after correction for multiple testing (FDR). For a given cluster, ethnic groups with the same letter did not present significant differences.

### Correspondence between the genetic structure and farmers’ variety names

The MMb cluster A was clearly separated from the others, as illustrated by the Neighbor-Joining tree. It included the four control individuals stemming from certified seeds of the *Gadam* improved variety, which has been disseminated in the area since 2009. Most of the other individuals assigned to cluster A were identified by farmers as *Gadam* (Chuka: 50%, Mbeere: 71%, Tharaka: 48%), confirming the cluster A – *Gadam* correspondence. Yet cluster A also included 46% of varieties collected under other names, mainly *Ngirigacha* (Chuka: 29%, Mbeere: 24%, Tharaka: 28%) and *Mbura-imwe* (Chuka: 11%, Tharaka: 16%). As a result, cluster A was distributed throughout the study area and its spatial distribution appeared more uniform than that of the individuals designated by farmers as *Gadam* (Figure 5.A & C).

The MMb cluster C was also clearly separated from the others, yet with an array of individuals that appeared as intermediates (along the branch of the Neighbor-Joining tree). The major share of the individuals assigned to cluster C was identified by farmers as an improved variety called *Kaguru*, which was introduced in the area about ten years ago (Chuka: 83%, Mbeere: 94%, Tharaka: 100%). *Kaguru* individuals originated uniformly from the study area (Figure 5.A) and in the three ethnic groups (Figure S1), but were less frequent in the Chuka area (Figure 5.C). The proportion of the Chuka sorghum individuals assigned to cluster C was significantly smaller (9%) as compared to the Tharaka (21%) population (Fisher test: p-value  = 0.023), and the Mbeere (25%) population (Fisher test: p-value  =  0.009). Half of the individuals (52%) collected under the name *Kaguru* in the Chuka farms were admixed, while this proportion was significantly lower in the Mbeere farms (15%, Pairwise Fisher test p-value: 0.0300) and in the Tharaka farms (10%, p-value  =  0.0190). Accordingly, the genetic diversity parameter estimates calculated for the *Kaguru* individuals collected in the Chuka farms were significantly higher than those for the Mbeere and Tharaka farms (Table 3). Altogether, these observations suggest that more admixture occurred between the *Kaguru* population and local landraces within the Chuka cropping systems than within the Tharaka and Mbeere systems.

**Table 3:** Summary of the genetic polymorphism indexes of the *Kaguru* variety sampled in the three ethnic groups.

Ethnic group	N_hh_	N_i_	*R_Al._*	*He*	*Ho*	*F_IS_*
**Chuka**	19	22	3.29 ^a^	0.339 ^a^	0.049	0.857
**Mbeere**	17	20	2.33 ^b^	0.184 ^b^	0.003	0.985
**Tharaka**	13	20	1.83 ^b^	0.091 ^c^	0.006	0.939

N_i_: number of samples, N_hh_: number of households, *R_Al_*: unbiased allelic richness. *H_e_*: unbiased gene diversity, *H_o_*: observed heterozygosity, *F_IS_*: fixation index. The letters (a, b, c) next to the *R_Al_* and *H_e_* values indicate the statistical significance of their differences between ethnic groups (Wilcoxon test) at a 5% level after correction for multiple testing (FDR). For a given index, ethnic groups with the same letter did not present significant differences.

The MMb cluster D appears clearly separated but rather heterogeneous on the Neighbor-Joining tree. On a morphological basis, these varieties mostly fall in a clearly distinct group. Most individuals assigned to cluster D were identified as long-cycle landraces by farmers (*Muruge mbura ciiri, Mugana*, *Muthigo*, *Mucuri*, *Kathirigwa)* and a few as short-cycle improved varieties (*Serendo* and *Musalama)*. The latter individuals identified as improved varieties, both collected on-farm and stemming from certified seeds, formed a distinct genetic sub-group D’ on the Neighbor-Joining tree and STRUCTURE confirmed these results for *K* = 5. The rest of the individuals assigned to cluster D were distributed across three major sub-clusters (Figure 4.A). Most *Muruge mbura ciiri* individuals clustered together in a separate branch on the Neighbor-Joining tree (D1). *Mugana* and *Kathirigwa* formed another branch (D2), and *Mucuri* a third one (D3). Hence, there was a clear correspondence between the farmers’ nomenclature and the genetic structure of individuals assigned to MMb cluster D, as well as with the structure of panicle morphological diversity (Figure S4). Cluster D was mainly observed in the Chuka area, as seen on Figure 5.C and confirmed with pairwise Fisher tests (p-value < 0.05). Interestingly, the few Tharaka households where we collected individuals assigned to cluster D were located in the Chuka area. Moreover, one household located on the eastern side presented several individuals assigned to cluster D, but it was a Chuka household settled in the Tharaka area (Figure 5).

The MMb cluster B is both central and diverse on the basis of molecular markers as well as morphological traits. The individuals assigned to cluster B were mainly identified as local landraces bearing various local names, whose occurrence frequency differed across ethnic groups (Table 4). Most of those collected in the Chuka and Tharaka farms were named *Muruge mbura imwe*, *Mugeta* and *Mbura imwe* while no or very few individuals collected in the Mbeere farms were named as such. Moreover, most of those collected in the Mbeere farms were named *Ngirigacha* (61%), while fewer individuals bore that name in the Chuka (8%) and Tharaka (18%) populations. Cluster B accounted for a uniformly large share among the farmers of the Tharaka (39%), the Chuka (27%) and the Mbeere (27%) ethnic groups. It showed little internal sub-structure with no clear correspondence to farmers' varieties, and a morphological differentiation between *Muruge mbura imwe* and *Mugeta* (Figure S4). As the only individuals with peculiar features, four individuals assigned to MMb cluster B for *K* = *4* formed a separate branch on the genetic Neighbor-Joining tree (B’) and their difference was confirmed by STRUCTURE for *K* = 5. It could be explained by their foreign origin, as farmers reported purchasing these seeds at a lowland market. A fifth individual assigned to cluster B for *K* = *4* formed a long branch (B”) indicative of a marked genetic differentiation. It was identified as *Muthigo wa mwimbi* which means that it was introduced from another ethnic group (*Mwimbi*).

**Table 4:** Proportion of individuals of each variety assigned to MMb cluster B regarding their collection ethnic group.

Variety	Chuka	Mbeere	Tharaka
**Muruge mbura imwe**	14 (39*%*)	1 (5*%*)	10 (29*%*)
**Mugeta**	10 (28*%*)	-	8 (24*%*)
**Ngirigacha**	3 (8*%*)	11 (61*%*)	6 (18*%*)
**Mbura imwe**	7 (19*%*)	-	8 (23*%*)
**Muthigo wa mwimbi**	1 (3*%*)	-	-
**Others :**			
**Gadam**	1 (3*%*)	2 (12*%*)	2 (6*%*)
**Kaguru**	-	1 (5*%*)	-
**Muruge mbura ciiri**	-	3 (17*%*)	-
**Total**	36 (100%)	18 (100%)	34 (100%)

Percentages in brackets.

## Discussion

Our study showed that in a uniform agro-ecological environment, social boundaries associated with ethnolinguistic diversity patterns have impacted the distribution of sorghum varieties and their genetic spatial patterns. If seeds, knowledge and practices were freely exchanged across the three ethnic groups, we would expect their sorghum varieties to be similar and, because of their geographical proximity and similar environmental conditions, to display no genetic differentiation. Quite the contrary, we showed that ethnic groups maintained different sorghum landraces, whereas improved varieties were uniformly distributed across groups.

### Factors structuring the distribution of sorghum genetic diversity

The genetic diversity of sorghum in the area of study, as assessed with molecular markers, is organized in four major groups. These groups reflected the influence of improved variety dissemination and a differentiation in terms of cycle duration and phenology. The improved varieties (groups A and C) and short-cycle landraces (group B) collected on the area of study clustered with the Caudatum accessions from eastern Africa and central Africa of a reference set representing the worldwide sorghum genetic diversity ([47]; Figure S3). The long-cycle landraces (group D) clustered with accessions from various origins (eastern & central Africa, India, Middle-East) and races (Durra, Caudatum, Bicolor and intermediates). Some new alleles, absent from the global reference set, were found in the local pool and notably among the long-cycle landraces, which could hence complement the reference set.

The overall distribution patterns of sorghum diversity on our study site were clearly associated with the farmers’ ethnic partition. This genetic differentiation did not appear to result from isolation-by-distance as no significant relationship between the geographic distance and the genetic relatedness of individuals was detected. Long-cycle landraces formed a genetically distinct cluster which was more frequently encountered in the Chuka sorghum population than in the Tharaka sorghum population. The improved *Kaguru* variety showed more admixture with the local landraces in the Chuka sorghum population than in the Mbeere and Tharaka ones. As a result of the unbalanced frequency of the different genetic clusters across ethnic groups, the genetic differentiation of their sorghum populations was significant. The uneven distribution of named landraces across the Chuka, Tharaka and Mbeere ethnic groups is consistent with the results of Baco et al. [48], who reported that different ethnic groups in Benin cultivated different varieties of yam. A similar relationship between the structure of the genetic diversity of domesticated populations and farmers’ social organization was found in taro populations across linguistic groups in Vanuatu [6], and in goat populations across ethnic groups in Vietnam [7]. However, a common caveat to such crop diversity studies conducted on large spatial scales is the difficulty involved in assessing whether the spatial patterns of crop diversity are related to variations in agro-ecological conditions, geographical distances, or to socio-cultural differences between human societies [10].

The field setting adopted in our study enabled us to limit the interference between socio-cultural factors and other environmental factors. Notably, climate and soil variations can influence the distribution of crop diversity. The climatic variation was neglectable on our study site regarding the limited gradient of altitude. In addition, we conducted a survey which did not highlight significant differences of soils’ physical properties among the areas inhabited by the three ethnic groups (data not shown). Furthermore, farmers did not report that some varieties were better adapted to particular types of soils. Hence, the interference between socio-cultural factors and other uncontrolled environmental factors remains much unlikely, even though it cannot be totally left out.

The community-scale approach we used in this study revealed that social boundaries have contributed to the differentiation of sorghum populations across spatially-close ethnic groups living in the same agro-ecological environment. Such an approach is thus complementary to country or regional-scale studies. In addition, such an approach makes it possible to investigate the mechanisms behind the relationship by jointly analyzing the distribution of varieties and the structure of genetic and morphological diversity in relation to the social organization of the communities concerned.

The ethnic identity of human groups is maintained by social boundaries that impede their cultural homogenization [49]. Our results suggest that these social boundaries also maintain differences between crop populations across ethnic groups. Indeed, gene flows in crop populations greatly depend on the exchange of seed, which is facilitated by social relationships and limited by social boundaries [4]. In addition, farmers’ seed selection practices have a strong impact on crop populations and can differ considerably across communities [5], [50]. The comparison of the structure of the genetic and morphological diversity of sorghum populations provides information concerning gene flows and selection forces, while the study of the nomenclature given to farmers’ varieties tracks the diffusion of knowledge across farming communities. Thus, by combining the two approaches it is possible to investigate the respective influence of seed exchanges and the diffusion of selection practices across ethnic groups on sorghum genetic diversity patterns.

### Limited diffusion of long-cycle landraces across ethnic groups

Long-cycle landraces formed a distinct MMb genetic cluster, whose frequency differed across ethnic groups. It was more frequent among the Chuka than among the Tharaka, and, interestingly, these results confirmed farmers’ reports stating that long-cycle landraces were “*Muvia wa Chuka*”, the sorghum of Chuka people. Moreover, certain sub-types within this cluster (sub-clusters D2 and D3) were not present in the Mbeere population and corresponding landraces were not inventoried in that ethnic group. The relation between the spatial distribution of the MMb genetic clusters and that of ethnic groups suggests that social boundaries limit the diffusion of planting material. Indeed, in most rural societies, seed exchanges depend on social networks as trust is required for seed transactions [51]. On the one hand, social relationships directly shape the seed exchanges because they facilitate access to seed [52], [53], [54]. On the other hand, the social network is the major pathway for information exchange [55] and indirectly helps shape seed exchanges, as farmers tend to imitate relatives [56]. The joint action of these two mechanisms can thus explain the uneven distribution of long-cycle landraces across ethnic groups. In addition, the small grains and the bitter taste could explain the low economic value of these landraces, which probably helps limit their diffusion.

### Management practices of improved varieties differ across ethnic groups

In contrast to the case of some landraces, improved varieties were uniformly distributed and their frequencies did not differ between ethnic groups. The recently introduced *Gadam* variety was genetically distinct from the landraces and showed limited introgression from the other genetic clusters. It was genetically uniform and complied with certified control. However, farmers also gave the names of local and already known variety to individuals that have the same genetic profile as *Gadam*, an improved variety. This can be explained by a morphological similarity. Yet it raises the question of the consequences this will have for the on-farm evolution of the improved variety. *Kaguru*, for instance, which was introduced in the area 10–15 years ago, seems to have evolved differently across ethnic groups. High admixture was detected between *Kaguru* and the local landraces in the Chuka population, resulting in a range of genetically diverse materials still called *Kaguru*, while this variety was found to be genetically more uniform in the Mbeere and Tharaka populations. As a result, the genetic diversity of *Kaguru,* as identified by farmers, was greater in the Chuka population than in the Mbeere and Tharaka populations. According to the farmers, the variety was introduced simultaneously in the three ethnic groups but little information is available concerning the origin of the seed lots. The divergence of the *Kaguru* variety across ethnic groups within a few decades could thus be the result of differences in their management practices, be it in planting (spatial arrangement of the varieties) or in seed selection. The higher admixture rate between *Kaguru* and the local landraces among the Chuka could be due to more intense gene flows within fields or to less stringent selection practices. As our observations suggest that the cropping systems used by the three ethnic groups were similar, the hypothesis of different selection practices is more likely. Cases of divergent selection practices between geographically close communities were observed by Pressoir and Berthaud [50], and by Perales et al. [5], who hypothesized that social boundaries impede the homogenization of selection practices. However, the hypothesis of the introduction of seed lots with different genetic characteristics in the three ethnic groups cannot be excluded.

### Divergence in the nomenclature of the landraces between ethnic groups

Comparing the genetic structure of short-cycle landraces, their morphological characteristics and the farmers’ nomenclature raises interesting questions concerning the relation between farmers’ nomenclature and the diffusion of planting material. Indeed, the frequencies of the majority of named short-cycle landraces differed significantly between ethnic groups even though they were assigned to the same genetic pool and no clear correlation was detected between named landraces and the MMb genetic sub-structure. The molecular markers used did not discriminate the three major short-cycle landraces whose frequency varied markedly across ethnic groups and which display different morphological characteristics. The short-cycle landraces grown by the different ethnic groups thus appeared to belong to the same genetic pool. Yet, the analysis of the morphological characteristics of the panicles suggests that the landraces presented morphological differences that were not detected with neutral genetic markers. *Mugeta* and *Muruge mbura imwe*, mainly grown by the Chuka and Tharaka, corresponded to two distinct morphological groups while *Ngirigacha*, which is mainly grown by the Mbeere, was distributed over the entire PCoA plot (Figure S4.A). These results suggest that ethnic groups use different names for landraces with similar morphotypes: the Chuka and Tharaka appear to identify and name two main short-cycle landraces corresponding to distinct morphotypes while the Mbeere mainly use the name *Ngirigacha* for all the morphotypes corresponding to the short-cycle landraces group.

This difference in folk-nomenclature and classification between the Mbeere and both the Chuka and Tharaka groups may result from limited knowledge diffusion. This is consistent with a number of observations concerning the conflictual relationship between the Chuka and Mbeere groups [19]. The impact of social relationships on the diffusion of folk-taxonomy and nomenclature among farming communities was demonstrated by Boster [57], who showed that the cassava nomenclature used by kin-related women was more similar than that used by non-kin in the Aguaruna community in Peru. Nuijten and Almekinders [12] also reported that the naming of rice varieties was more consistent within villages than between villages in Gambia. They pointed out that information concerning varieties, such as names, is not necessarily passed on with the seed lots. Hence, seed exchanges between communities can be more intense than knowledge diffusion, leading to the use of different names for similar morphotypes and genotypes. Further comparison of farmers’ nomenclature and taxonomy between ethnic groups is required to confirm this hypothesis.

Previous studies showed that different sorghum varieties may display no genetic differentiation despite being morphologically distinct. Notably, in Cameroon, Barnaud et al. [3] showed that considerable gene flows existed between Guinea sorghum landraces while farmers kept on selecting them for their morphological distinctiveness. Rabbi et al. [58] reported similar results in western Kenya, while varieties collected in eastern Sudan were clearly genetically distinct. He explained these results by the varietal isolation practiced in Sudan, while Kenyan farmers mixed varieties within their fields. Soler et al. [59] found that landraces were distinct genetic units, but in that study each landrace was sampled in a single field belonging to one farmer, which considerably limits the variability. As farmers’ taxonomy and nomenclature is based on morphological traits with a simple genetic determinism, morphological differences can be maintained even though gene flows occur within farmers’ fields. The 18 SSRs used in our study were selected because they revealed high polymorphism in previous diversity studies, and they proved to be adequate for characterizing the genetic sub-structure of long-cycle landraces. However, their resolution power may not be sufficient to reveal a finer-scale genetic sub-structure in short-cycle landraces. The use of high-density markers may help to evidence finer-scale genetic structure and could hence contribute to decipher the evolutionary mechanisms that molded the landraces.

### Effect of community social organization on the diffusion of seeds, knowledge and farmers’ practices

According to local elders, the three ethnic groups migrated to the study area about a century ago. Our results suggest that even though they have lived in proximity since then, the way knowledge, practices and seeds are diffused has maintained differences between sorghum populations across ethnic groups. Ethnographic observations of community social organization provide explanations for such limited exchanges across geographically close communities. Indeed, information transmission and diffusion appear to be confined within the residential groups (parents and married sons) first, which is common in patrilineal and patrilocal societies [60], and next within the neighborhood group, which is a major social institution among eastern Kenyan Bantu communities [16], [61], (Linsig pers.com). The way knowledge is transmitted and diffused is very conservative and favors cultural differentiation between communities [62], [63]. It thus probably plays a major role in maintaining differences in nomenclature and practices between ethnic groups, and maybe also in limiting seed exchanges.

## Conclusion

Our study highlights the importance of local short-scale studies to investigate farm crop evolution processes. To date, emphasis has been placed on the effect of agro-ecological conditions on crop evolution processes, as in the study of the evolution of wild plants. The influence of the cultural diversity and social organization of farming communities has consequently been neglected, although the major role of smallholders in the management of crop diversity has been acknowledged [64]. Crop evolution is still ongoing in smallholder farming systems and such systems occupy a substantial proportion of croplands in developing countries, especially in Africa [65]. Most of these rural communities have retained pre-colonial social institutions that continue to shape the relationships between people. Sixty-eight living language groups were inventoried in Kenya and about 2146 linguistic groups in Africa [66], so the situation of ethnic co-existence described in this paper is not an isolated case. This study confirms the influence of the ethnolinguistic patterns of rural communities on gene flows and on farmers’ selection practices that shape crop diversity *in situ*. Crop diversity patterns, thus result not only from an interaction between genetic and environmental factors, G × E, but from a three-way interaction G × E × S, where ‘‘S’’ stands for effects of the social boundaries [4]. Investigating this relation in other communities, with different social organizations and rules for the transmission of knowledge, would thus help gain a clearer picture of crop evolution dynamics in subsistence farming systems.

A further study is now needed to probe the mechanisms involved. Notably, the link between seed exchange networks and social organization deserves more investigation to confirm whether seed exchanges are confined within ethnic groups. This would explain why the diffusion of long-cycle landraces is more limited than that of short-cycle landraces. Moreover, further comparison of the local sorghum nomenclature and classification systems (folk taxonomy) across ethnic groups would make it possible to test whether their definition of landraces differs, and whether it influences their seed selection practices.

The uneven distribution of the genetic clusters across ethnic groups within a restricted geographic area highlights the need to take the social relationship and exchanges into account in the characterization, collection, and conservation of crop diversity. Accounting for the impact of human practices on crop populations would help capture their diversity more efficiently and, to this end, ethnic contact zones are of major interest for their potentially high genetic diversity. This study paves the way for participatory plant breeding as it shows that farmers’ individual choices concerning planting material are not only determined by agro-ecological conditions or economic interest, but also by their cultural background.

## Supporting Information

Figure S1 **Diagram displaying the rain seasons and the growth-cycle of sorghum on our study site.** Inventories’ dates are symbolized by the letter I (orange points) and collections’ dates by the letter C (red points).
(TIF)

Figure S2 **Comparison between the inventory of varieties and their sampling.** (A) Percentage of households where each variety was sampled for the genetic diversity study on a total of 130 households. (B) Linear correlation between the proportions of households where each variety was inventoried (vertical axis, 124 households) and where it was collected (horizontal axis, 130 households) in each ethnic group.
(TIF)

Figure S3 **Neighbor-Joining tree based on the genetic dissimilarity among the individuals sampled on our study site (in Black) and the accessions of a global reference set (Billot et al. 2013).** The genetic dissimilarities were calculated on 16 SSRs using the simple matching index. The sorghum individuals sampled on our study site are displayed in black. The genetic assignment (A, B, C, D - q>0.8) or unassignment (Unassigned - q≤0.8) of our individuals is indicated on the figure. Colors represent the ten genetic groups identified in Billot et al. 2013, and described as following by the authors: “Group 1 [Dark orange] included Caudatum, Caudatum-Bicolor and Durra from Eastern Asia; Group 2 [Light orange] encompassed Durra and Bicolor from the Indian subcontinent, while Group 3 [Light green] exhibited Durra from Eastern Africa. Bicolor and Durra-Bicolor from Eastern Africa were assigned in Group 4 [Light blue]. Group 5 [Dark blue] included Guinea and Guinea margaritiferum from Western Africa and Bicolor from North America. Group 6 [Red] appeared as a well-separated group made predominantly of Guinea accessions from western Africa, accompanied by intermediate race Durra-Caudatum materials from western Africa while Group 7 [Magenta] was made essentially of materials collected from eastern Africa and central Africa generally classified as race Caudatum (visible along FA axis 3). Group 8 [Dark green] was a small and heterogeneous group made of Durra and Caudatum race accessions from central Africa. Group 9 [Pink] was made essentially of Guinea race accessions from the Indian subcontinent and southern/eastern Africa with Guinea-Caudatum (GC) intermediate race accessions from various parts of Africa. Group 10 [Purple] was made almost exclusively of accessions from southern Africa of race Kafir or intermediate race Kafir-Caudatum (KC).” Unassigned individuals in the global reference set are displayed in grey.
(TIF)

Figure S4 **Structure of the morphological and genetic diversity within the MMb clusters B (top) and D (bottom).** (A) Plot of the two first axes of the Principal Coordinates Analysis (PCoA) done on the sorghum plants assigned to the MMb cluster B and based on 15 panicle morphological traits. The first axis (x) expresses 35.1% of the total variation and the second axis 13.1%. Varieties are displayed using the following color code: Blue: *Mugeta*, purple: *Muruge mbura imwe*, green: *Mbura imwe*, brown: *Ngirigacha*, Red: *Gadam*, yellow: *Kaguru*, salmon: *Muthigo wa mwimbi*. (B) Neighbor-Joining tree based on the genetic dissimilarity among individuals assigned to the MMb cluster B calculated on 18 SSRs using the simple matching index. (C) Plot of the two first axes of the Principal Coordinates Analysis (PCoA) done on the sorghum plants assigned to the MMb cluster D and based on 15 panicle morphological traits. The first axis (x) expresses 51.5% of the total variation and the second axis 18.0%. Varieties are displayed using the following color code: Yellow: *Serendo*, orange: *Musalama*, light-pink: *Kathirigwa*, Fushia: *Mugana*, Greenish blue: *Mucuri*, dark-blue: *Muruge mbura ciiri*, black: *Muthigo*, blue: *Mugeta*. (D) Neighbor-Joining tree based on the genetic dissimilarity among individuals assigned to the MMb cluster D calculated on 18 SSRs using the simple matching index.
(TIF)

Table S1 **Summary of the sampling of planting material.** Mean number of varieties collected per household (Mean no. varieties/household) and mean number of samples of each variety collected per household (Mean no. samples/variety/household) in each ethnic group, followed by their standard error (SE).
(DOCX)

Table S2 **Summary of information and genetic diversity estimates per locus.** Minimum and maximum size of alleles (Size), chromosome where the locus is located (Ch), percentage of missing data per locus (Miss), number of sampled alleles (N_Al_), *He*: unbiased gene diversity, *F_IS_*: Fixation index.
(DOCX)

Table S3 **Morphological descriptors used for panicle description.**
(DOCX)

Table S4 **Results from the perMANOVA comparing the effect of ethnic groups on sorghum variety assemblages.** Df: degrees of freedom, Ssq: sequential sum of squared distance between individuals and their group’s centroïd, Mean Ssq  =  Ssq/Df, F.Model: pseudo F ratio, R^2^: coefficient of determination [Ssq Etnic group/Ssq Total].
(DOCX)
