# Supplementary figures and images for: Influence of Ethnolinguistic Diversity on the Sorghum Genetic Patterns in Subsistence Farming Systems in Eastern Kenya
Source: PLoS One. 2014 Mar 17;9(3):e92178. doi: 10.1371/journal.pone.0092178 (PMC3956919; doi:10.1371/journal.pone.0092178)

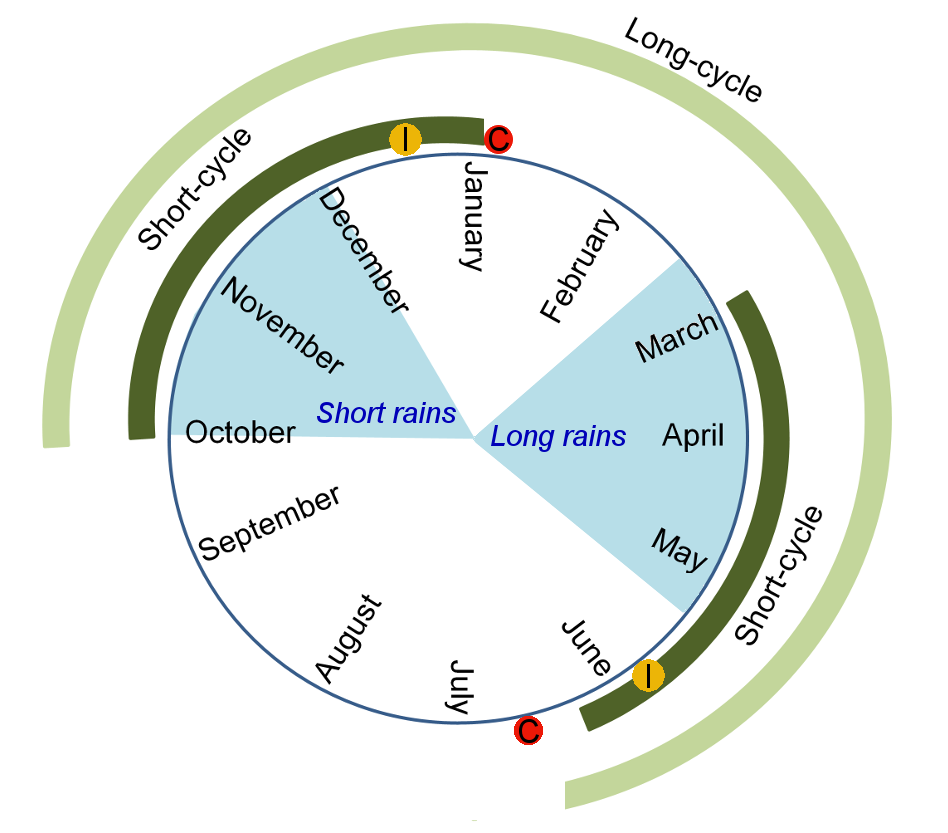

Supplement: Figure S1 — Diagram displaying the rain seasons and the growth-cycle of sorghum on our study site. Inventories’ dates are symbolized by the letter I (orange points) and collections’ dates by the letter C (red points). (TIF) [file pone.0092178.s001.tif]

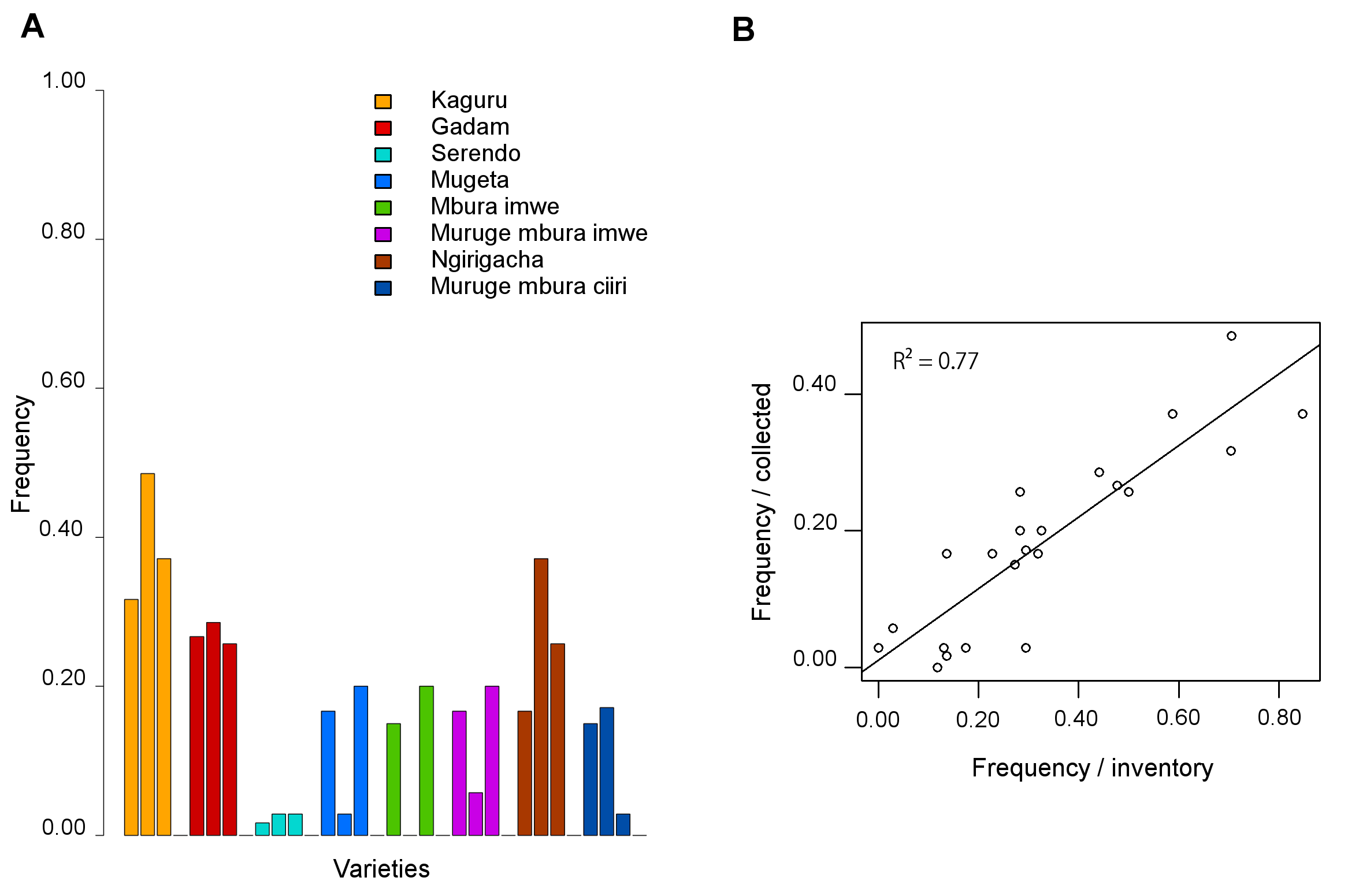

Supplement: Figure S2 — Comparison between the inventory of varieties and their sampling. (A) Percentage of households where each variety was sampled for the genetic diversity study on a total of 130 households. (B) Linear correlation between the proportions of households where each variety was inventoried (vertical axis, 124 households) and where it was collected (horizontal axis, 130 households) in each ethnic group. (TIF) [file pone.0092178.s002.tif]

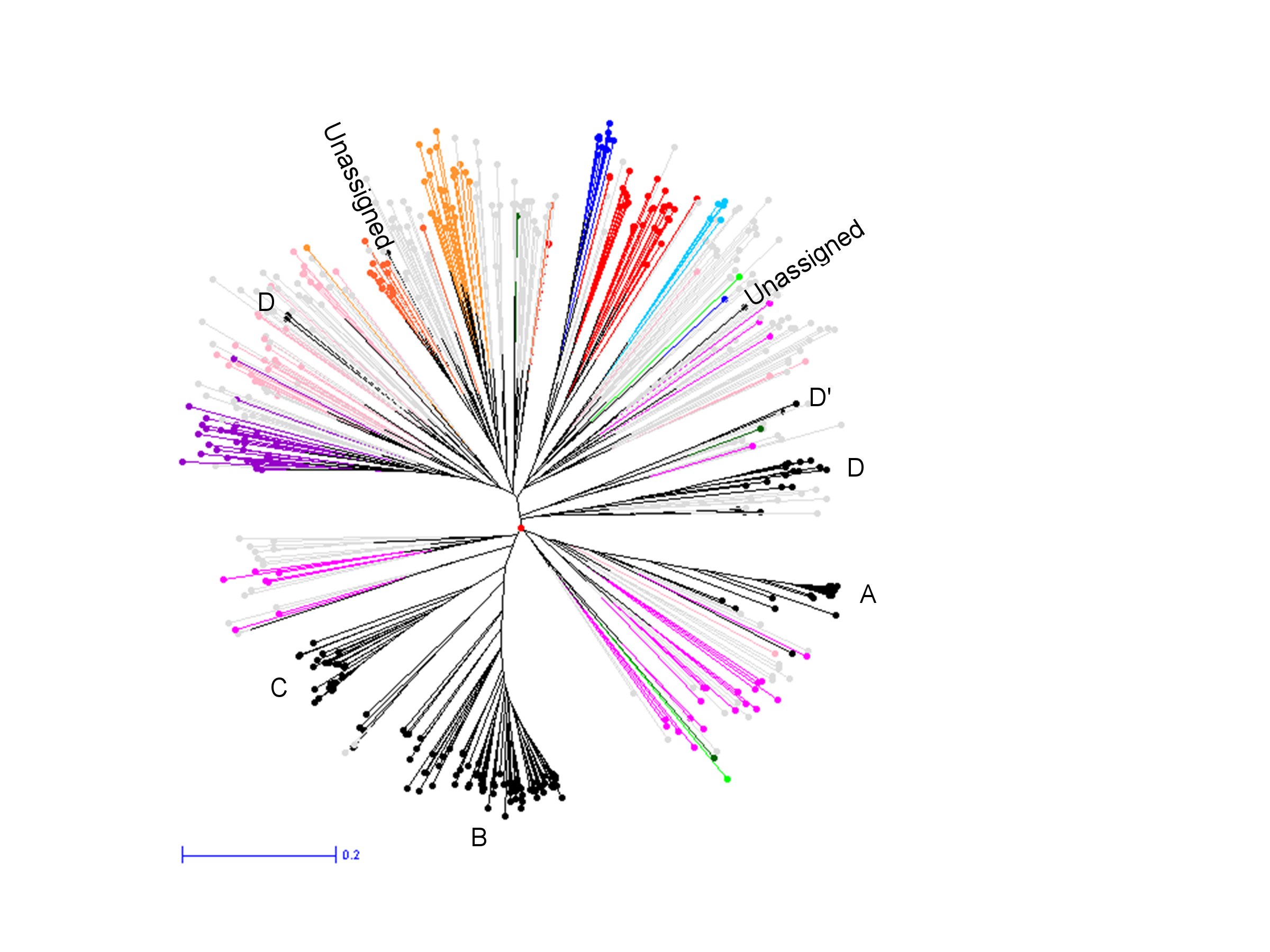

Supplement: Figure S3 — Neighbor-Joining tree based on the genetic dissimilarity among the individuals sampled on our study site (in Black) and the accessions of a global reference set (Billot et al. 2013). The genetic dissimilarities were calculated on 16 SSRs using the simple matching index. The sorghum individuals sampled on our study site are displayed in black. The genetic assignment (A, B, C, D - q>0.8) or unassignment (Unassigned - q≤0.8) of our individuals is indicated on the figure. Colors represent the ten genetic groups identified in Billot et al. 2013, and described as following by the authors: “Group 1 [Dark orange] included Caudatum, Caudatum-Bicolor and Durra from Eastern Asia; Group 2 [Light orange] encompassed Durra and Bicolor from the Indian subcontinent, while Group 3 [Light green] exhibited Durra from Eastern Africa. Bicolor and Durra-Bicolor from Eastern Africa were assigned in Group 4 [Light blue]. Group 5 [Dark blue] included Guinea and Guinea margaritiferum from Western Africa and Bicolor from North America. Group 6 [Red] appeared as a well-separated group made predominantly of Guinea accessions from western Africa, accompanied by intermediate race Durra-Caudatum materials from western Africa while Group 7 [Magenta] was made essentially of materials collected from eastern Africa and central Africa generally classified as race Caudatum (visible along FA axis 3). Group 8 [Dark green] was a small and heterogeneous group made of Durra and Caudatum race accessions from central Africa. Group 9 [Pink] was made essentially of Guinea race accessions from the Indian subcontinent and southern/eastern Africa with Guinea-Caudatum (GC) intermediate race accessions from various parts of Africa. Group 10 [Purple] was made almost exclusively of accessions from southern Africa of race Kafir or intermediate race Kafir-Caudatum (KC).” Unassigned individuals in the global reference set are displayed in grey. (TIF) [file pone.0092178.s003.tif]

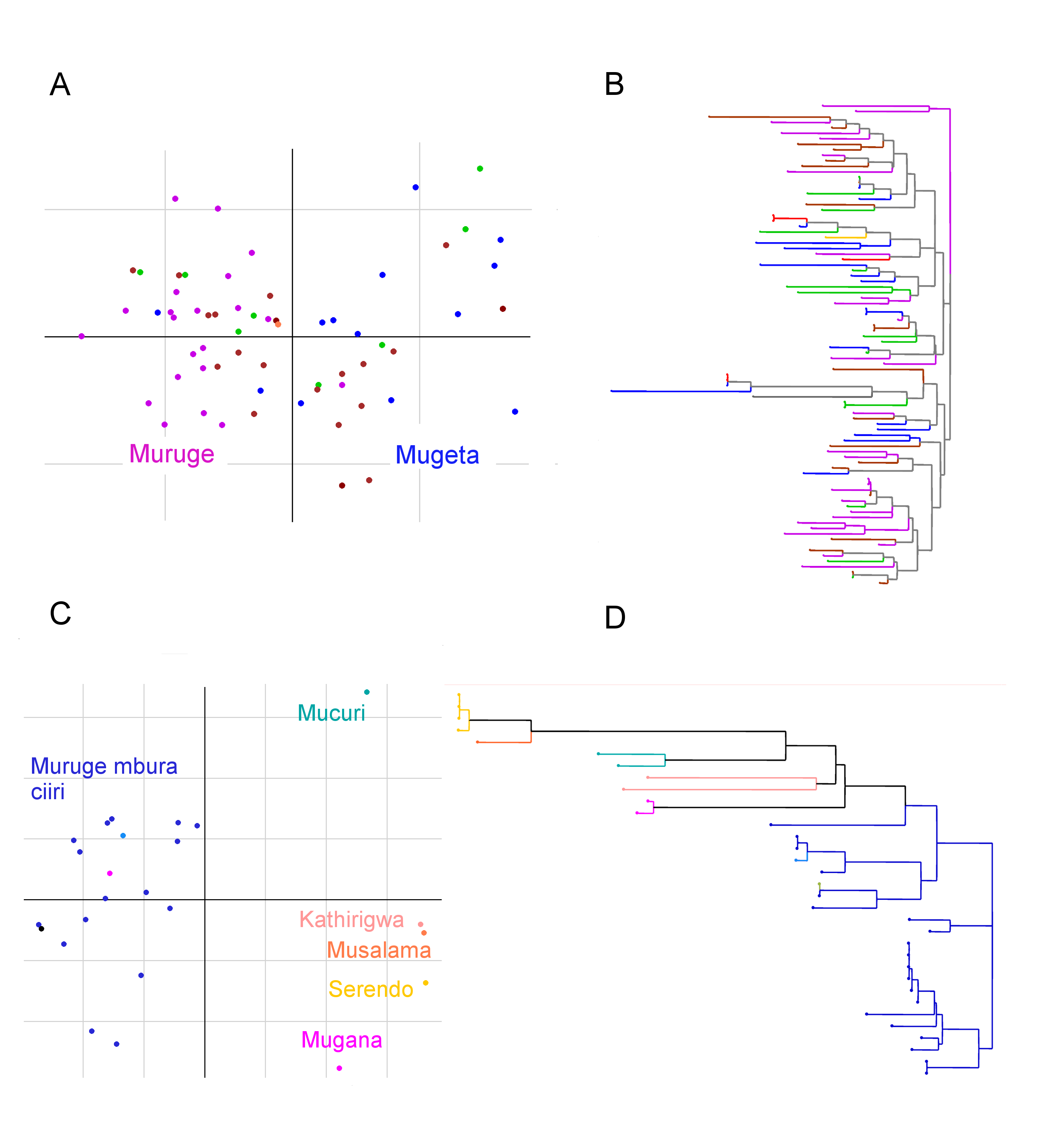

Supplement: Figure S4 — Structure of the morphological and genetic diversity within the MMb clusters B (top) and D (bottom). (A) Plot of the two first axes of the Principal Coordinates Analysis (PCoA) done on the sorghum plants assigned to the MMb cluster B and based on 15 panicle morphological traits. The first axis (x) expresses 35.1% of the total variation and the second axis 13.1%. Varieties are displayed using the following color code: Blue: Mugeta, purple: Muruge mbura imwe, green: Mbura imwe, brown: Ngirigacha, Red: Gadam, yellow: Kaguru, salmon: Muthigo wa mwimbi. (B) Neighbor-Joining tree based on the genetic dissimilarity among individuals assigned to the MMb cluster B calculated on 18 SSRs using the simple matching index. (C) Plot of the two first axes of the Principal Coordinates Analysis (PCoA) done on the sorghum plants assigned to the MMb cluster D and based on 15 panicle morphological traits. The first axis (x) expresses 51.5% of the total variation and the second axis 18.0%. Varieties are displayed using the following color code: Yellow: Serendo, orange: Musalama, light-pink: Kathirigwa, Fushia: Mugana, Greenish blue: Mucuri, dark-blue: Muruge mbura ciiri, black: Muthigo, blue: Mugeta. (D) Neighbor-Joining tree based on the genetic dissimilarity among individuals assigned to the MMb cluster D calculated on 18 SSRs using the simple matching index. (TIF) [file pone.0092178.s004.tif]
