# Supplementary material for: Influence of Ethnolinguistic Diversity on the Sorghum Genetic Patterns in Subsistence Farming Systems in Eastern Kenya
Source: PLoS One. 2014 Mar 17;9(3):e92178. doi: 10.1371/journal.pone.0092178 (PMC3956919; doi:10.1371/journal.pone.0092178)
Supplement: Table S1 — Summary of the sampling of planting material. Mean number of varieties collected per household (Mean no. varieties/household) and mean number of samples of each variety collected per household (Mean no. samples/variety/household) in each ethnic group, followed by their standard error (SE). (DOCX) [file pone.0092178.s005.docx]

Table S1. Summary of the sampling of planting material. Mean number of varieties collected per household (Mean no. varieties / household) and mean number of samples of each variety collected per household (Mean no. samples / variety / household) in each ethnic group, followed by their standard error (SE).

|  | **Chuka** | **Mbeere** | **Tharaka** |
| --- | --- | --- | --- |
| Mean No. varieties / household | 1.67 [SE: 0.14] | 1.46 [SE: 0.13] | 1.71 [SE: 0.20] |
| Mean No. samples / variety / household | 1.51 [SE: 0.06] | 1.32 [SE: 0.07] | 1.56 [SE: 0.08] |
