# Supplementary material for: Influence of Ethnolinguistic Diversity on the Sorghum Genetic Patterns in Subsistence Farming Systems in Eastern Kenya
Source: PLoS One. 2014 Mar 17;9(3):e92178. doi: 10.1371/journal.pone.0092178 (PMC3956919; doi:10.1371/journal.pone.0092178)
Supplement: Table S2 — Summary of information and genetic diversity estimates per locus. Minimum and maximum size of alleles (Size), chromosome where the locus is located (Ch), percentage of missing data per locus (Miss), number of sampled alleles (NAl), He: unbiased gene diversity, FIS: Fixation index. (DOCX) [file pone.0092178.s006.docx]

Table S2. Summary of information and genetic diversity estimates per locus. Minimum and maximum size of alleles (Size), chromosome where the locus is located (Ch), percentage of missing data per locus (Miss), number of sampled alleles (N_Al_), *He*: unbiased gene diversity, *F_IS_*: Fixation index.

| **SSR Marker** | **Size (pb)** | | **Ch** | **Miss** | ***N_Al._*** | ***He*** | ***F_IS_*** |
| --- | --- | --- | --- | --- | --- | --- | --- |
|  | **min** | **max** |  |  |  |  |  |
| mSbCIR223 | 120 | 140 | 2 | 0% | 3 | 0.125 | 1 |
| mSbCIR248 | 100 | 120 | 5 | 1% | 4 | 0.051 | 0.932 |
| sb4-72 | 196 | 235 | 6 | 1% | 3 | 0.401 | 0.913 |
| sb5-206 | 121 | 174 | 9 | 1% | 13 | 0.774 | 0.951 |
| sb6-84 | 192 | 240 | 2 | 0% | 6 | 0.58 | 0.94 |
| sbAgb02 | 111 | 179 | 7 | 1% | 5 | 0.519 | 0.967 |
| xcup02 | 199 | 229 | 9 | 0% | 4 | 0.699 | 0.941 |
| xcup14 | 224 | 256 | 3 | 0% | 4 | 0.472 | 0.971 |
| xcup53 | 200 | 230 | 1 | 0% | 3 | 0.503 | 0.945 |
| xcup61 | 214 | 223 | 3 | 0% | 2 | 0.493 | 0.937 |
| xtxp10 | 140 | 174 | 9 | 0% | 8 | 0.73 | 0.962 |
| xtxp12 | 180 | 230 | 4 | 4% | 13 | 0.679 | 0.968 |
| xtxp21 | 180 | 220 | 4 | 4% | 13 | 0.76 | 0.976 |
| xtxp289 | 270 | 346 | 5 | 2% | 11 | 0.667 | 0.947 |
| xtxp295 | 150 | 210 | 7 | 0% | 13 | 0.753 | 0.949 |
| xtxp320 | 271 | 304 | 1 | 1% | 10 | 0.723 | 0.908 |
| xtxp321 | 160 | 180 | 8 | 1% | 11 | 0.685 | 0.975 |
| xtxp57 | 240 | 276 | 6 | 2% | 13 | 0.742 | 0.957 |
