# Supplementary material for: Influence of Ethnolinguistic Diversity on the Sorghum Genetic Patterns in Subsistence Farming Systems in Eastern Kenya
Source: PLoS One. 2014 Mar 17;9(3):e92178. doi: 10.1371/journal.pone.0092178 (PMC3956919; doi:10.1371/journal.pone.0092178)
Supplement: Table S3 — Morphological descriptors used for panicle description. (DOCX) [file pone.0092178.s007.docx]

Table S3. Morphological descriptors used for panicle description.

| **Morphological descriptors** | **No. of modalities** | **Modalities** |
| --- | --- | --- |
| Panicle shape | 8 | Broom; Very loose; Loose; Semi-loose; Semi-compact long; Semi-compact ; Compact elliptic; Very compact |
| Grain shattering | 2 | Mid; High |
| Seed color | 5 | White; Cream; Grey; Brown; Red |
| Seed shape | 2 | Asymetric; Non-asymetric |
| Pericarp thickness | 2 | Thin; Thick |
| Subcoat | 2 | Present; Absent |
| Endosperm texture | 2 | Mainly vitreous, Mainly floury |
| Glume adherence | 3 | High; Mid; Low |
| Glume opening | 4 | Half-open; Highly open; Mid; Tight |
| Glume covering | 2 | Full; Mid |
| Awn | 2 | Present; Absent |
| Glume transversal wrinkle | 2 | Present; Absent |
| Glume texture | 2 | Hard; Papery |
| Glume color | 3 | Black; Red; Tan |
| Glume hairiness | 2 | Mid; High |
