# Supplementary material for: Influence of Ethnolinguistic Diversity on the Sorghum Genetic Patterns in Subsistence Farming Systems in Eastern Kenya
Source: PLoS One. 2014 Mar 17;9(3):e92178. doi: 10.1371/journal.pone.0092178 (PMC3956919; doi:10.1371/journal.pone.0092178)
Supplement: Table S4 — Results from the perMANOVA comparing the effect of ethnic groups on sorghum variety assemblages. Df: degrees of freedom, Ssq: sequential sum of squared distance between individuals and their group’s centroïd, Mean Ssq = Ssq/Df, F.Model: pseudo F ratio, R2: coefficient of determination [Ssq Etnic group/Ssq Total]. (DOCX) [file pone.0092178.s008.docx]

Table S4. Results from the perMANOVA comparing the effect of ethnic groups on sorghum variety assemblages. Df: degrees of freedom, Ssq: sequential sum of squared distance between individuals and their group’s centroïd, Mean Ssq = Ssq/Df, F.Model: pseudo F ratio, R^2^: coefficient of determination [Ssq Etnic group / Ssq Total]

|  | **Df** | **SSq** | **Mean SSq** | **F.Model** | **R^2^** | **Pr(>F)** |
| --- | --- | --- | --- | --- | --- | --- |
| **Ethnic group** | 2 | 2.047 | 1.023 | 4.971 | 0.076 | 0.0002* |
| **Residuals** | 121 | 24.912 | 0.206 | 0.924 |  |  |
| **Total** | 123 | 26.959 | 1 |  |  |  |
